# Kinematic Zenith Tropospheric Delay Estimation with GNSS PPP in Mountainous Areas

**DOI:** 10.3390/s21175709

**Published:** 2021-08-25

**Authors:** Paul Gratton, Simon Banville, Gérard Lachapelle, Kyle O’Keefe

**Affiliations:** 1 Position Location and Navigation (PLAN) Group, Department of Geomatics Engineering, Schulich School of Engineering, University of Calgary, 2500 University Drive NW, Calgary, AB T2N 1N4, Canada; lachapel@ucalgary.ca (G.L.); kpgokeef@ucalgary.ca (K.O.); 2 Canadian Geodetic Survey, Natural Resources Canada, 588 Booth Street, Ottawa, ON K1A 0Y7, Canada; simon.banville@canada.ca

**Keywords:** GNSS, PPP, ZTD, zenith tropospheric delay, VMF1

## Abstract

The use of global navigation satellite systems (GNSS) precise point positioning (PPP) to estimate zenith tropospheric delay (ZTD) profiles in kinematic vehicular mode in mountainous areas is investigated. Car-mounted multi-constellation GNSS receivers are employed. The Natural Resources Canada Canadian Spatial Reference System PPP (CSRS-PPP) online service that currently processes dual-frequency global positioning system (GPS) and Global’naya Navigatsionnaya Sputnikovaya Sistema (GLONASS) measurements and is now capable of GPS integer ambiguity resolution is used. An offline version that can process the above and Galileo measurements simultaneously, including Galileo integer ambiguity resolution is also tested to evaluate the advantage of three constellations. A multi-day static data set observed under open sky is first tested to determine performance under ideal conditions. Two long road profile tests conducted in kinematic mode are then analyzed to assess the capability of the approach. The challenges of ZTD kinematic profiling are numerous, namely shorter data sets, signal shading due to topography and forests of conifers along roads, and frequent losses of phase lock requiring numerous but not always successful integer ambiguity re-initialization. ZTD profiles are therefore often only available with float ambiguities, reducing system observability. Occasional total interruption of measurement availability results in profile discontinuities. CSRS-PPP outputs separately the zenith hydrostatic or dry delay (ZHD) and water vapour content or zenith wet delay (ZWD). The two delays are analyzed separately, with emphasis on the more unpredictable and highly variable ZWD, especially in mountainous areas. The estimated delays are compared with the Vienna Mapping Function 1 (VMF1), which proves to be highly effective to model the large-scale profile variations in the Canadian Rockies, the main contribution of GNSS PPP being the estimation of higher frequency ZWD components. Of the many conclusions drawn from the field experiments, it is estimated that kinematic profiles are generally determined with accuracy of 10 to 20 mm, depending on the signal harshness of the environment.

## 1. Introduction

Global navigation satellite systems (GNSS) precise point positioning (PPP) is used routinely to estimate the zenith tropospheric delay (ZTD) and its components at static sites worldwide to study lower atmospheric layers and contribute to the understanding of weather patterns and prediction. PPP theory and ZTD application to weather literature is voluminous and excellent examples are [1,2,3,4]. The International GNSS Service (IGS) publishes discrete ZTD values for its worldwide stations [5]. An IGS Troposphere Working Group coordinates its activities, e.g., [6]. Methods of implementing GNSS tropospheric delay estimates in weather analysis are described in detail in [7].

ZTD is strongly correlated with height above sea-level. The zenith dry (hydrostatic) delay (ZHD) is predictable accurately given atmospheric pressure measurements at the receiver location, using Saastamoinen’s ZHD model [8]. However, the zenith wet delay (ZWD) is less predictable and more variable. ZHD is of the order of 2.0 m at sea level and decreases with increasing elevation/decreasing atmospheric pressure. ZWD is determined by the highly variable water vapor and temperature in the troposphere above the receiver, the latter impacting the amount of water the atmosphere can sustain. Surface pressure, temperature, and humidity can be easily measured in both static and kinematic testing, but the humidity is highly variable as a function of elevation above the observation point, making the use of such surface measurements unreliable to estimate 3D ZWD profiles [2].

ZTD is estimated in GNSS PPP using a combination of spatial GNSS measurements and surface weather data to model the atmospheric vertical profile. The estimated ZTD parameter is separated from other parameters estimated by PPP as it is the only parameter which is dependent on the satellite elevation angle above the horizon in the formulation of the PPP observation equation, given by [9] as:

(1) $${\bar{L}}_{g,m,f}^{S}=-e_{x}\cdot \delta x+dT_{g}+\zeta_{w}^{S}\cdot \delta T_{Z}+\zeta_{G}^{S}\cdot \left[G_{N}\cos\left(a\right)+G_{E}\sin\left(a\right)\right]-\mu_{g,f}\cdot I^{S}+\lambda_{g,f}N_{g,m,f}^{S}+b_{g,f}$$

(2) $${\bar{C}}_{g,m,f}^{S}=-e_{x}\cdot \delta x+dT_{g}+\zeta_{w}^{S}\cdot \delta T_{Z}+\zeta_{G}^{S}\cdot \left[G_{N}\cos\left(a\right)+G_{E}\sin\left(a\right)\right]-\mu_{g,f}\cdot I^{S}+B_{g,f}$$

for uncombined carrier (L) and code (C) measurements, where the overbar symbol indicated misclosures. The superscript *S* indicates that a different parameter is given for each satellite, while the subscripts *g*, *m*, and *f* indicate different parameters for different constellations, modulations, and frequency bands, respectively. The direction cosine vector between the satellite and receiver is given as $e_{x}$ and the correction to the a priori receiver coordinates is given as δx. Each frequency band carrier wavelength is given as $\lambda_{g,f}$. The receiver clock offset ($dT_{g}$), the phase ambiguity ($N_{g,m,f}^{S}$), and code and phase biases ($B_{g,f}$ and $b_{g,f}$) each have constant partial derivatives; therefore, additional constraints described by [9] and the datum ambiguity method of integer ambiguity resolution introduced by [10] are applied to eliminate rank deficiencies. The ionospheric delay for each satellite ($I^{S}$) is separable due to its frequency dependent multiplier $\mu_{g,f}=F_{g,1}^{2}/F_{g,f}^{2}$, where $F_{g,f}$ is the frequency of carrier *f* and $F_{g,1}$ is always the frequency of one of the carriers; hence, $\mu_{g,1}=1$. Therefore, observations on at least two frequency bands are necessary for the most accurate removal of ionospheric effects. While other ionospheric correction methods for single-frequency data exist such as the group and phase ionospheric calibration (GRAPHIC) [2] or an empirical ionospheric model, these are not as accurate as the use of dual-frequency observations. In Equation (1), the ZHD is considered removed by modelling, and the remaining ZWD term is given as $\delta T_{Z}$, with the corresponding wet mapping function $\zeta_{w}^{S}$. North and east gradients ($G_{N}$ and $G_{E}$) are also estimated to account for equatorial atmospheric bulge and azimuthal (a) variations caused by weather systems [2]. The tropospheric gradients have a separate mapping function $\zeta_{G}^{S}$.

Functions that map slant measurements to the local zenith are used to estimate ZTD with good accuracy, a prime example being VMF1, the Vienna Mapping Function 1 [11]. For high-accuracy PPP solutions, it is necessary to separate the ZHD and ZWD components due to the differences in hydrostatic and wet mapping functions at lower elevation angles. PPP software generally estimates and outputs ZTD and its two components, ZHD and ZWD. Typically, the more predictable ZHD values are modelled, while the remainder is estimated as ZWD. The separate estimation of ZWD parameters is valuable for numerical weather prediction (NWP) algorithms as it can be used to determine water vapour content in the troposphere above the receiver. The two-sigma accuracy estimates of IGS-derived ZTD values at IGS stations using continuous 24 h data segments are of the order of 2 mm to 8 mm and a function of various factors, e.g., regional satellite geometry and environmental obstructions at the station sites.

**Figure 1:**
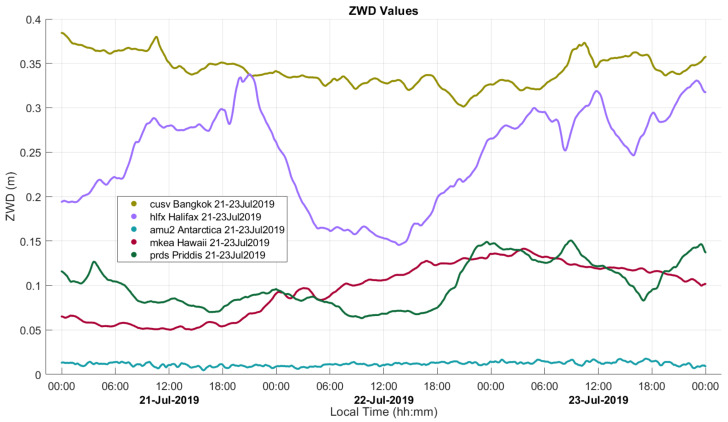
ZWD variability at five IGS stations. Sea-level heights are approximately 100 m (Bangkok), 25 m (Halifax), 2845 m (Antarctica), 3725 (Hawaii), and 1265 m (Priddis).

The spatial and temporal variability of ZWD is illustrated in Figure 1 using observation data from five permanent tracking stations, four operated by the IGS and one (AMU2) operated by UNAVCO (formerly University NAVSTAR Consortium), processed with Canadian Spatial Reference System PPP (CSRS-PPP) software, developed by Natural Resources Canada (NRCan) in Ottawa, Canada. The lowest and most stable profile is predictably at the Amundsen-Scott south pole station (AMU2) at 2845 m above sea level during the Antarctic winter when extreme cold temperature cannot sustain any significant water vapour. The highest profile shown is for Bangkok near sea level, where the high temperature and humidity result in ZWD values of 30 to 37 cm. The Halifax, Canada station is also near sea level by the Atlantic Ocean but in a more temperate zone with highly variable weather, resulting in variations of 15 to 34 cm over a three-day period during summer. Priddis provides the closest comparison of ZWD behaviour near the mountainous area of interest, with a relatively high elevation at 1265 m and low humidity creating a low profile with maximum values at 15 cm. The profile for Hawaii, near the top of the Mauna Kea volcano, shows similar values to those at Priddis, as the extreme elevation at 3725 m is balanced by the relatively high humidity of the tropical Hawaiian climate.

The prime objective of this paper is to assess the feasibility of PPP to estimate zenith tropospheric delays along road profiles in mountainous areas using GNSS equipment mounted on a vehicle, hence ZTD kinematic PPP estimation. While the estimation of ZTD with static GNSS data has been well examined, kinematic ZTD estimation has not been investigated thoroughly. While several previous studies have been performed with similar goals, for example [12,13], these have several key differences to the current study. The vehicular kinematic data used by [12] was on a single trajectory with limited GNSS obstructions. The current study differs in that multiple trajectories are used with varying levels of GNSS obstruction, which was found to significantly affect the results. Additionally, only GPS was used by [12], as the Galileo constellation did not exist and GLONASS was not ready for this type of investigation. While [13] performed repeated tests along a mountainous kinematic trajectory with an elevation gain of 950 m, the same trajectory was repeated multiple times; hence, the same level of obstruction was present in each case, assuming a similar satellite distribution throughout. Again, testing in the current research was performed using trajectories with widely varying levels of obstruction. The study in [13] also did not use the Galileo constellation, again as it was not operational at that time. Neither [12] nor [13] used low-cost, dual-frequency receivers, due to unavailability at those times. The final significant factor separating [12,13] from the current study is the use of recently developed PPP with carrier phase integer ambiguity resolution, termed PPP-AR. The software used for this research does implement PPP-AR, while the other studies did not. Once integer ambiguities are fixed to integers, they can be removed from the estimated parameters, thus improving the observability of all remaining parameters, including the ZTD. In this study, the accuracy of the ZWD is evaluated by considering both the consistency of ZWD estimation repeatability between receivers, combined with the uncertainty of ZHD models used to separate the two ZTD components. In [12], a Radiometrics (Boulder, Colorado) WVR-1100 water vapour radiometer (WVR) was used, which was able to determine integrated liquid water and water vapour along a selected path (for example the zenith path), at a static point, which was used as truth data for GNSS-derived ZWD values along kinematic trajectories within 5 km of the WVR. Integrated liquid water and water vapour measurements can be used to determine ZWD as described by [7]. The use of a WVR was not possible for kinematic tests with long horizontal distances travelled and height variations over 1000 m as was carried out for the current study. Truth data for [13] were derived by interpolation of ZWD estimates at static stations at each end of the single kinematic trajectory used. This approach also could not be applied to this study as multiple kinematic trajectories were used along very different routes.

Mountain weather is notorious for its rapid spatial and temporal variability [14], hence the interest in focusing on such an area. The major limitations of kinematic PPP estimation in mountains versus the static approach are (1) the presence of unpredictable multipath and changing obstructions along the trajectories due to the forestry canopy and mountainous topography, both resulting in frequent losses of phase lock and sub-optimal carrier phase integer ambiguity resolution; (2) data sets of up to several hours instead of days for static measurements at permanent stations; and (3) high correlation with epoch-by-epoch position estimates, rather than a single static position. The advantage is the ability to observe zenith tropospheric delays along profiles of changing elevations, 1200 to 2200 m in the present case, and the effect of changing mountain weather conditions. An extreme example of this benefit would be in the use of ZWD estimates from aircraft during ascent and descent as analyzed by [12]; however, this was beyond the scope of the current study. The primary receivers used for this purpose are high-end geodetic units. A sub-objective is to compare their performance to lower cost units under the conditions described above. The software used is the online Natural Resources Canada CSRS-PPP software service which accepts global positioning system (GPS) and Global’naya Navigatsionnaya Sputnikovaya Sistema (GLONASS) (GR) measurements at this time and attempts to resolve GPS carrier phase ambiguities as integers [9]. Offline developmental CSRS-PPP software is also used to process combined GPS, GLONASS and Galileo (GRE) measurements.

Numerous tests were first conducted in static mode to assess methodology, equipment, and software, in addition to analyzing repeatability between the above receivers operating at the same site. One of these static tests is first examined prior to proceeding to the analysis of two kinematic tests, many more of which were also conducted and are described in [15]. The use of both static and kinematic tests enables one to examine performance differences between static and kinematic approaches.

## 2. Equipment, Software and Field Testing

### 2.1. Equipment

In the results and analyses presented in Section 3, two types of receivers are used, namely (1) the Leica (Heerbrugg, Switzerland) GS16 geodetic-grade receiver, and (2) the u-blox (Thalwil, Switzerland) ZED-F9P, which was mounted on the u-blox C099-F9P application board for simplicity of use. As a high-grade receiver, the high consistency of the GS16 is well-known. The capability of the lower-cost ZED-F9P for tropospheric delay estimation in static mode was recently demonstrated by [16]. The GS16 receivers simultaneously track L1 C/A, L2W and L2C(M) signals in the GPS L1 and L2 bands, GLONASS L1 C/A and L2 C/A signals, and Galileo E1, E5a, E5b, and AltBOC signals. The u-blox ZED-F9P receiver tracks GPS L1 C/A and modernized L2C signals, GLONASS L1 C/A and L2 C/A signals, and Galileo E1 B/C and E5b signals. To account for the ZED-F9P use of GPS L2C signals, the CSRS-PPP software must exclude GPS Block IIR satellites that do not broadcast the L2C signal and could therefore only be observed with single-frequency observations. During data collection, 31 GPS satellites were operational, eight of which were Block IIR satellites. Between data collection and the time of writing, one Block IIR satellite (PRN G28) was decommissioned on 21 June 2021. The current status is available online at https://www.navcen.uscg.gov/?Do=constellationStatus (accessed on 13 July 2021).

Two or three receivers were used in each test to assess consistency. Two GS16 receivers labelled GS16B and GS16R and one F9P receiver labelled F9PBLU were used. The GS16 antenna is housed internally in the receiver housing. The F9P test kit came with a low-cost u-blox ANN-MB-00 antenna whose calibration parameters were not available to the authors. Early tests with this antenna versus a geodetic grade NovAtel (Calgary, Canada) GPS-703-GGG antenna showed more consistent results with the latter, which was therefore used for the tests presented below. It is, however, possible to overcome the low-cost antenna limitation by performing a relative antenna calibration with nearby precise receiver-antenna systems operating in static mode, as demonstrated by [16]; this was outside the scope of this study. GPS and GLONASS L1 and L2 antenna calibrations were available and applied in the PPP software for both the GS16 and NovAtel GPS-703-GGG antennas. Correct orientation of the antenna north reference point (NRP) is recommended due to anisotropic gain patterns of antennas which are accounted for in the antenna calibration files. This cannot be maintained during kinematic tests along roads with many changes in direction and it can impact solution accuracy [17]. A static test was conducted with a deliberately incorrect orientation to estimate the magnitude of the effect. The consistency of height and ZWD estimates between receivers were not degraded compared to tests with correctly oriented antennas. Antenna calibration parameters for Galileo are not currently available; therefore, the GPS antenna calibration parameters were applied for Galileo signals. As the GPS L1 and Galileo E1 signals are in the same frequency band, it is likely that the difference between GPS L2 and Galileo E5 frequency bands presents the most issue with the use of GPS calibrations for Galileo signals. No impact was, however, detected in the ZTD and height results of the three-constellation solutions presented in Section 3. The integer ambiguity resolution success was somewhat different between online software GR solutions and developmental software GRE solutions, as will be shown in Section 3. It appears that these differences are due to the difference in ephemeris products between the online and developmental versions (rather than the Galileo constellation or antenna calibrations), as when GR solutions are produced with the developmental version, they are more consistent with the GRE solutions. An example of this behaviour is described in the analysis of kinematic results.

**Figure 2:**
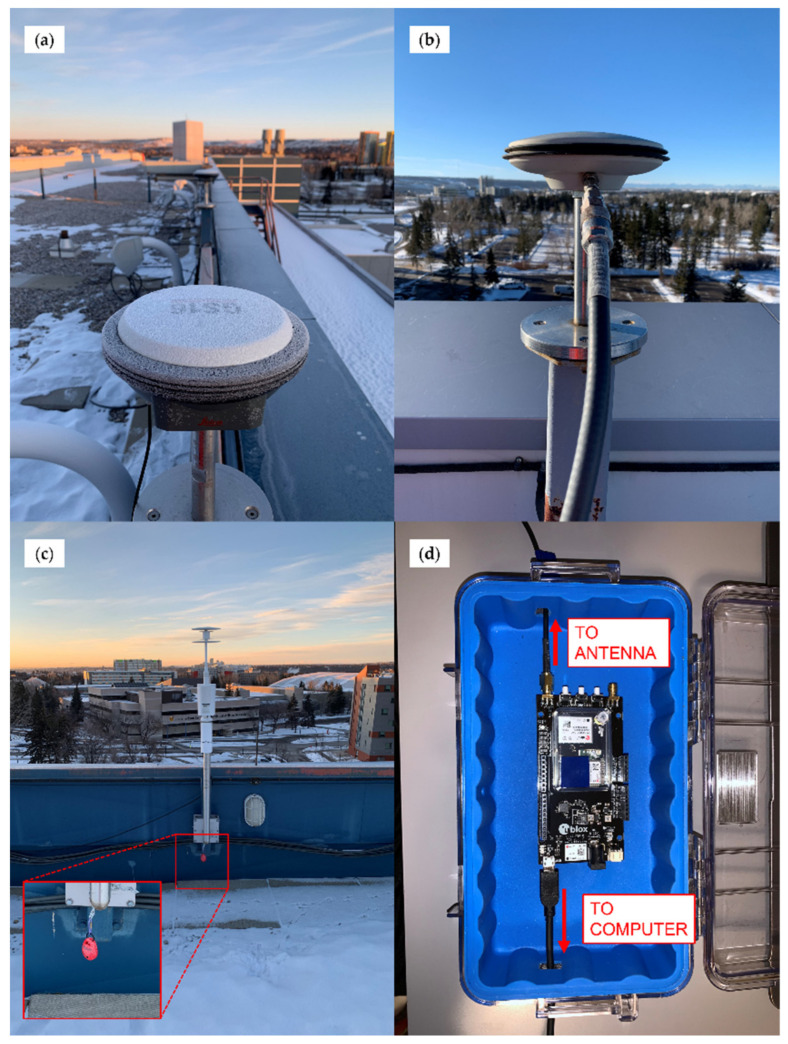
Equipment used for testing consisted of: (**a**) GS16R (near) and GS16B (far) receivers installed on permanent pillars for static testing; (**b**) NovAtel GPS-703-GGG antenna connected to antenna cable network for static testing; (**c**) Kestrel DROP D3 environmental data logger installed in shaded location of static test location; (**d**) C099-F9P board of F9PBLU receiver in protective case used for kinematic testing indicating antenna and computer connections.

GS16 observations were logged directly by the receiver, operating on firmware version 8.00 (654) and measurement engine firmware 7.500 (0). F9P measurements were recorded by a Panasonic Toughbook laptop using the u-blox u-center v19.10 software package, with receivers operating on firmware version HPG 1.12. For static tests on a roof top, the F9P units were inside the building and connected to the roof antenna via a permanent antenna cable network. Images of the GS16 receivers, the F9PBLU receiver, and NovAtel GPS-703-GGG antenna are shown in Figure 2, as well as a Kestrel DROP D3 environmental data logger used during testing, as described in Section 2.3. During static and kinematic testing, the antennas were mounted between 0.8 m and 10 m of each other.

### 2.2. Weather Information

Surface atmospheric observations were collected using battery-operated portable Kestrel DROP D3 environmental data loggers to measure and show the variability of the weather during testing and determine the extent of correlation with ZWD variability. In each test, they were installed near one of the receivers. The unit specifications state the observation accuracy as ±0.5 °C in ambient temperature, ±2% in relative humidity, and ±1.5 hPa in air pressure. The inter-unit performance was verified with simultaneous observations with multiple units. RMS errors for all observed parameters was well within the specifications, with the maximum absolute errors only slightly exceeding the specifications (e.g., 3% maximum relative humidity difference between units). The Kestrel measurements were also compared with values produced by the permanent weather station at the Calgary International Airport 10 km away. Given likely small changes between the two sites, the values were found to be in excellent agreement.

A combination of surface and weather balloon atmospheric measurements could be used to predict both ZHD and ZWD through vertical weather profiles and mapping functions, the latter to relate slant GNSS measurements to vertical reference values. While measuring the pressure and height at each point is the most accurate method to predict ZHD using models such as Saastamoinen’s, the use of a 3-D pressure model generated from observations in the surrounding region can be used to approximate ZHD with accuracy on the order of 1 cm. Likewise, ZWD can also be predicted with surface measurements and appropriate 3D profiles, although, in this case, predictability is lower due to the high variability of humidity vertical profiles and higher ZWD frequency variations are not detected. Numerous vertical profile models and mapping functions are available for the above predictions, the most common being the Vienna Mapping Function 1 (VMF1) used here. VMF1 is maintained by TU Wien (Technische Universität Wien) and uses its own mapping functions and generates gridded (2.0° lat × 2.5° lon resolution) ZHD and ZWD values using vertical pressure, temperature, and humidity profiles provided by the European Centre for Medium-Range Weather Forecasts (ECMWF) as described by [11]. The ECMWF uses atmospheric weather stations from around the world, the locations of which can be seen on the World Meteorological Organization (WMO) website at: https://oscar.wmo.int/surface/index.html#/ (accessed on 13 July 2021). The coverage of surface stations around Calgary is good but decreases as one moves into the Rocky Mountains west of the city. Coverage of radiosonde weather balloon stations is highly limited, with only 38 stations in Canada. Of these 38 stations, only one is in Alberta, near Edmonton, and two are in British Columbia on the west side of the Rocky Mountains. Balloon launches only occur twice daily at each station, limiting the temporal resolution of the data. The VMF1 grid data are only updated every six hours and change within these periods cannot therefore be captured effectively. Height differences between VMF1 grid values and receiver heights are accounted for using the methodology described by [18]. In kinematic mode, these corrections also account for the significant changes in elevation experienced by the receiver as vehicular tests travel through the mountains. The use of this height correction model is analyzed later in the results shown in Section 3.3.1. The 3 mm/√hr process noise applied to the estimated ZWD parameter [9] is also analyzed.

**Figure 3:**
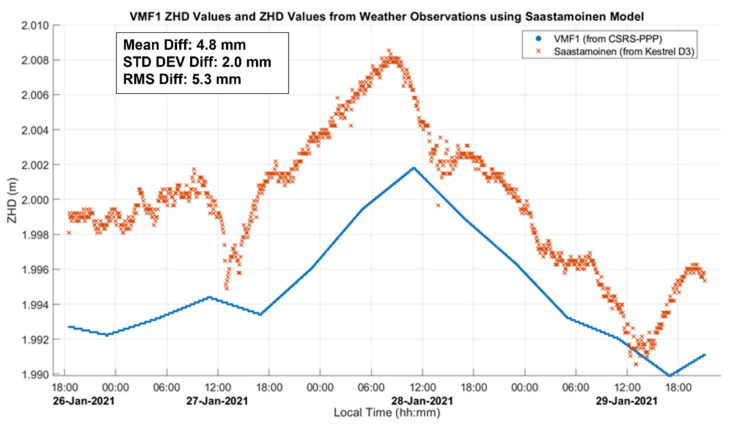
Static dataset A ZHD values produced by the CSRS-PPP using the VMF1 model over a three-day period and compared with values obtained using Saastamoinen’s model and Kestrel unit weather parameters. The six-hour updates of the VMF1 model are evident. The ZHD differences (Saastamoinen’s–VMF1) reach 8 mm and reflect local weather (mostly pressure) variations during the six-hour intervals. Local time is UTC−7:00.

CSRS-PPP computes the ZHD profile using VMF1 and the GNSS-derived heights output by the software itself. The smoothing effect of this profile for the three-day static test (dataset A) analyzed in Section 3.2 is shown in Figure 3 by comparing it with the ZHD profile derived directly using the continuous Kestrel pressure measurements with Saastamoinen’s model. Differences reach 8 mm due to the departure of the actual atmospheric pressure from the value predicted by VMF1, which is within the capabilities of the VMF1 ZHD model as shown by [11], wherein a maximum 16 mm bias from Saastamoinen’s model was reached in an analysis of global stations. The atmospheric variation during the dataset A 3-day period departed from the normal value of 889.7 hPa by 10 to 12 hPa, with each 10 hPa of variation at that elevation resulting in a 20 mm difference in the ZHD [11]. The above maximum difference of only 8 mm shows that VMF1 was able to predict some of the actual pressure at the point using regional atmospheric weather stations, with some remaining bias and high-frequency variations. CSRS-PPP also uses VMF1 as a priori information for ZWD, with the remainder estimated as a parameter in the PPP software. Surface atmospheric measurements with the Kestrel units were used to assess the ability of VMF1 to estimate ZHD values under the field conditions encountered but were not used in the CSRS-PPP software, which is not programmed to take such measurements as input.

### 2.3. Software 

CSRS-PPP v3.50.0 online implementation can process dual-frequency PPP solutions with GPS L1 and L2 and GLONASS L1 and L2 frequency bands. The service automatically selects the optimal pair of L1 and L2 signal modulations for dual-frequency PPP. Integer ambiguity resolution is only attempted for GPS signals, GLONASS ambiguities being estimated as float values. A developmental version of CSRS-PPP not yet available online uses in this case the Galileo E1 and E5a (for the GS16) or E5b (for the F9P) signals and attempts to fix the ambiguities as integers as well on these measurements. The results presented below are with GPS and GLONASS (GR), and with the three constellations (GRE) where indicated. The precise products used for GR solutions is a combination of the IGS Final orbit products for GPS and precise orbit products produced by NRCan for GLONASS. Clock products and observable-specific signal biases are produced in-house by NRCan, as described by [19]. For GRE solutions, GFZ (Deutsches GeoForschungs Zentrum) precise products are used for all constellations, with signal biases produced by Centre National d’Etudes Spatiales (CNES) in Paris, France using the methods given by [20], available online at http://www.ppp-wizard.net/products/POST_PROCESSED/ (accessed on 13 July 2021). CSRS-PPP sets a satellite elevation angle cutoff of 7.5°. During the tests documented herein, the cut-off selected for the equipment was generally 10°, a difference that is negligible in this case given obstructions along kinematic profiles. A new estimation method was implemented for version 3 of CSRS-PPP, termed sequential normal stacking, as described by [9]. Solutions are first processed forwards without resolving ambiguities as integers, then processed again backwards to implement integer ambiguity resolution. In static mode, a third run, forwards again, is performed as new information is added by the integer ambiguities. A back substitution of parameters allows a recovery of consistent final values of all parameters, including the position and ZWD estimates. This back substitution method, and the backwards processing of kinematic solutions which smooths the results is given by [9]. The standard deviations of code and carrier observations are given as 0.6 m and 0.0035 m, respectively, at the zenith, and multiplied by $1/\sin\left(e\right)$, where e is the elevation angle of the satellite above the horizon [9]. GLONASS inter-frequency code biases (IFCBs) are removed by a running average of GLONASS code residuals. Mis-modelling of GLONASS IFCBs is accounted for by de-weighting GLONASS code observations by a factor of 2. Standard deviations of Galileo observations are identical to those of GPS. Initial constraints and process noises of all estimated parameters are given by [9]. All solutions presented here are processed using data at 30 s intervals to avoid interpolation errors associated with satellite clock corrections. The ZHD and ZWD values are output explicitly at these intervals and can be simply added to produce the ZTD. Static sessions were also processed in kinematic mode to assess the level of agreement with static solutions.

**Figure 4:**
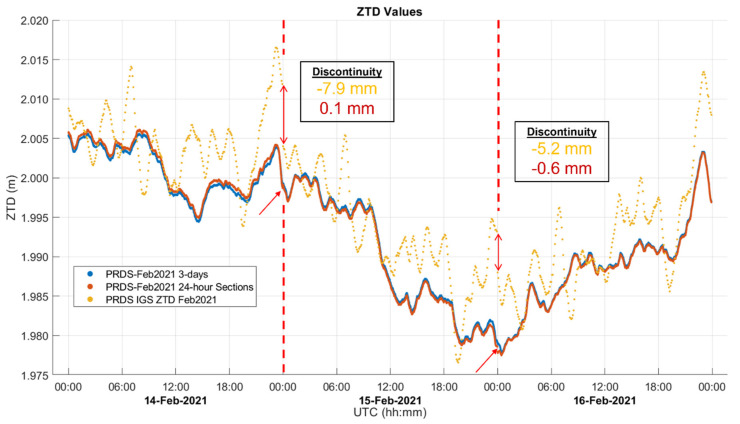
ZTD comparison between IGS-derived values (yellow) and CSRS-PPP (v3.50.0) continuous (blue) and 24 h (red) profiles over three days at IGS PRDS site.

An earlier version of CSRS-PPP (v1.05) that did not attempt to resolve carrier phase ambiguities as integers was compared with three other software packages using measurements available at selected IGS sites worldwide in [21]. Using ZTD values available on the IGS website as a reference, the differences between CSRS-PPP and IGS values range were found to be in the range of 1 to 2 cm for continuous measurement periods of 24 h. This level of agreement was similar to that of other software tested. The performance of the current version 3.50.0 was assessed using the same approach. Five three-day segments of measurements available from the PRDS IGS site 25 km south of Calgary and covering the 16-month period from early 2020 to April 2021 were reprocessed and ZTD results compared with the online IGS values. GPS integer carrier phase ambiguities were resolved over 98% of the time. The results for the five periods were similar and the ones for 14–16 February 2021 are shown in Figure 4. IGS ZTD values are derived using the Bernese software, developed at the Astronomical Institute of the University of Bern (AIUB) in Bern, Switzerland, with continuous datasets of 24 h; more information is provided by [6]. The three-day dataset was processed twice with CSRS-PPP, namely in a single segment of three days and in three segments of one day corresponding to the three segments used by IGS. The IGS ZTD values are discontinuous by up to 8 mm at the 24 h data segment junction points, while the corresponding numbers for CSRS-PPP are below 1 mm. Differences between the ZTD values produced by CSRS and IGS, but exceed 15 mm at a few points. Due to the back substitution performed by CSRS-PPP, the discontinuities that occur between 24 h segments are not caused by ambiguity convergence time at the beginning of each solution. This is the same for the IGS ZTD solutions in which case the Bernese software performs batch adjustments as described by [22]. The discontinuities are therefore likely due simply to random differences between independent solutions. The continuous CSRS-PPP 72 h solution does not experience any discontinuity.

### 2.4. Testing

**Figure 5:**
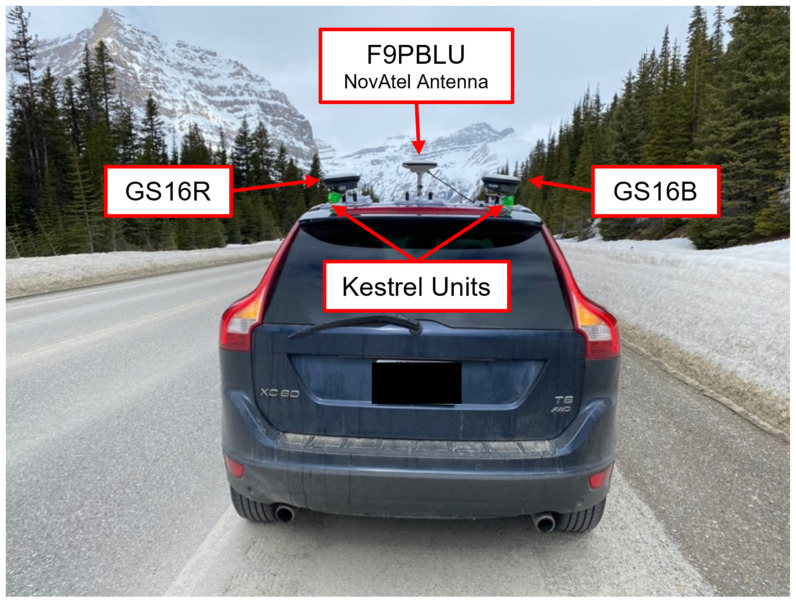
Typical configuration of GNSS receivers for kinematic testing. The Leica GS16 receivers are seen mounted to the rear of the roof on the left and right sides. The NovAtel antenna is mounted to the front center of the roof, connected to the F9P unit located inside the vehicle. Kestrel environmental data loggers are mounted to the magnet mounts of the GS16 receivers. (Photo taken at the Bow Pass, 2100 m elevation on the Icefield Parkway on 28 April 2021).

Field testing included static tests in Calgary and vehicular kinematic tests between Calgary and the nearby Rocky Mountains. Static testing was performed on the rooftop of the Calgary Centre for Innovative Technology (CCIT) building on the University of Calgary main campus. The CCIT building provides near-open-sky observing conditions and has permanent GNSS pillars with precisely known coordinates. All kinematic tests began in the Calgary area at 1000 m to 1200 m elevation and travelling west towards the Rocky Mountains, with some tests reaching maximum elevations of 2200 m. Figure 5 shows the antenna configuration on the vehicle used. The following three tests, labelled dataset A to C, are analyzed in the paper:

A. Static testing on the roof of the CCIT building over a period of 3 days (26–29 January 2021) using the full life of the Leica GEB371 external batteries. The F9PBLU receiver was connected to a NovAtel GPS-703-GGG antenna via permanent antenna cables that led into the building.
B. Kinematic testing from South Calgary to the Highwood Pass in Kananaskis Country via Hwy 22, Hwy 541 and Hwy 40 and return the same way, a total trajectory of 290 km, on 26 October 2020. No overpasses were present along the route taken. This trajectory contains both near-complete open-sky conditions and more obstructed conditions in mountainous areas. Two GS16 receivers were used for this test.
C. Kinematic testing on Hwy 1, Hwy 40, and Hwy 742 from Calgary to Chester Lake at an elevation of 1900 m in Kananaskis Country on 12 March 2020 and return, a total trajectory of 280 km. Obscuring overpasses were present. The forestry canopy, being close to the road, created additional continuous signal reception challenges. All three receivers were used.

## 3. Data Processing and Analysis

When analyzing tropospheric delay estimates of GNSS signals through PPP, the estimated receiver heights must also be analyzed as these parameters are correlated. Previous studies have shown that an error in ZTD modelling creates an error in height about 2.2 times the magnitude of the ZTD error (e.g., [23]). The correlated error in height would have the opposite sign of the ZTD error; hence, as height increases, ZTD decreases. In a PPP algorithm in which ZTD is estimated rather than modelled, either parameter can influence the other, depending on the way each is modelled.

In the analyses presented in the following sub-sections, the estimated ZTD and ZWD profiles are both provided. This is because the main contribution of GNSS measurements is in the estimation of the ZWD component and its correlation with height errors, while the ZTD is shown to provide context for the magnitude of the ZWD relative to the total tropospheric effect. GNSS measurements contribute to the ZHD components through providing positions and heights to VMF1 for which an accuracy of a few metres is sufficient. The estimated ZHD profile is therefore identical for receivers operating within several metres of each other as carried out here. As mentioned previously during the discussion of Figure 3, ZHD errors occur when the actual atmospheric pressure at the receiver is different from the interpolated values from the VMF1 grid. ZHD modelling errors cause errors in height estimates due to the difference in satellite elevation angle mapping functions for ZHD and ZWD parameters as described by [11]. The magnitude of ZHD errors in the dataset analyses is estimated by comparing the ZHD profile obtained with the pressure measurement profile obtained at the receivers using the Kestrel unit with Saastamoinen’s model and the corresponding VMF1-derived ZHD profile. These errors are somewhat compensated for in the ZWD estimation process. While this compensation preserves the positioning accuracy of the PPP solution, it is detrimental to the ZWD parameter accuracy itself, if it were to be used for numerical weather prediction purposes.

### 3.1. Receiver Code and Carrier Measurement Accuracy Analysis

**Figure 6:**
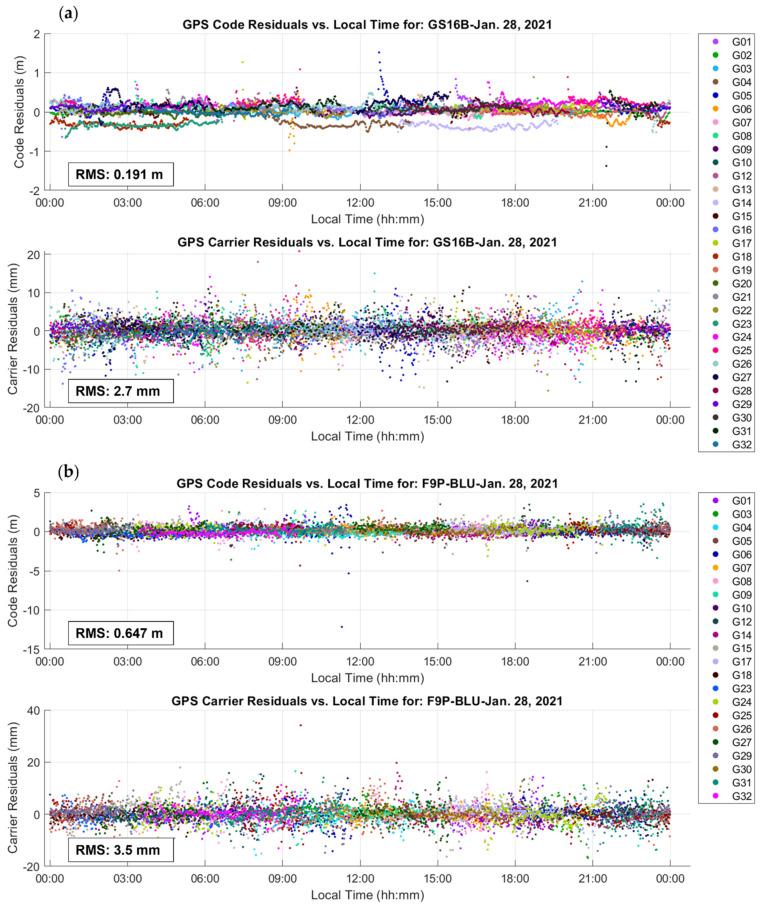
GPS L1 C/A code and carrier residuals of static PPP solution for 24 h of dataset A for: (**a**) GS16B receiver; (**b**) F9PBLU receiver using a NovAtel antenna. Vertical scales are different for each receiver. The F9P receiver shows fewer satellites as L2C is the only signal tracked in the GPS L2 frequency band; therefore, block IIR satellites (which do not broadcast the newer L2C signal) are not included in the dual-frequency PPP solution for this purpose. Local time is UTC−7:00.

The measurement accuracy of the receivers and antennas used in the tests are characterized by the GPS L1 C/A code and carrier residuals shown in Figure 6 for a 24 h period of dataset A. RMS values are also given in each plot for the residuals of each measurement type. The code RMS values range from 19 cm for the GS16B to 65 cm for the F9PBLU. Carrier RMS values range from 2.7 mm for the GS16B to 3.5 mm for the F9PBLU. Therefore, if PPP both integer ambiguity resolution is largely successful for both receiver types, the solution quality will be comparable, unless receiver or antenna biases occur, or the satellite geometry is significantly different when GPS Block IIR satellites are excluded from F9P solutions. If the resolution success rate decreases, float ambiguity quality and, by extension, solution quality, will depend partly on code accuracy, decreasing the consistency between receivers. The effect of removing GPS Block IIR satellites was tested on static dataset A, by removing them from observation files for both GS16 receivers before processing in PPP. Estimated heights were affected by approximately 4 mm, and height and ZWD 2σ uncertainties increased by approximately 0.5 mm. GPS Block IIR satellites were not removed from GS16 observations for the analyses provided in the following subsections to maintain the best possible performance level of the GS16 receivers.

### 3.2. Static Dataset “A” Analysis

**Figure 7:**
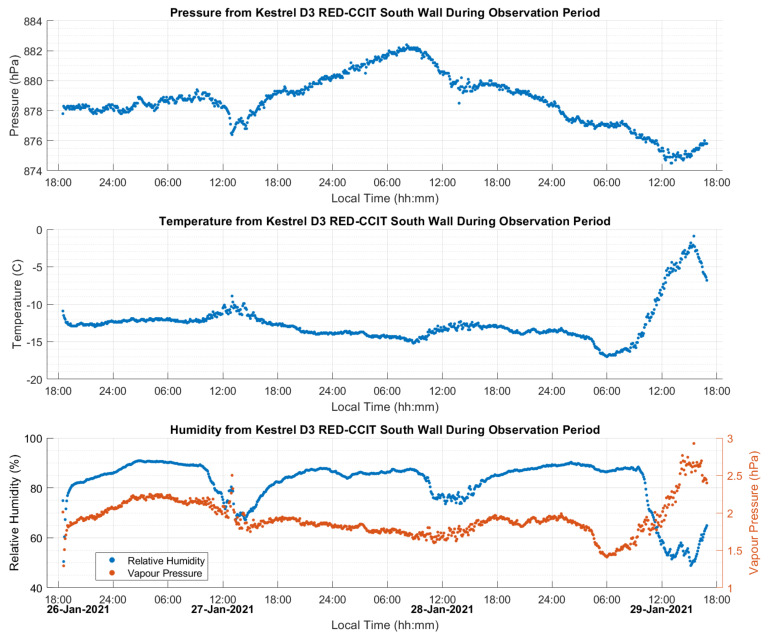
Static dataset A atmospheric conditions at test site (Elevation of 1135 m). Normal atmospheric pressure at 1135 m is 889.7 hPa. Local time is UTC−7:00.

Figure 7 shows the measured weather conditions at the site. Relatively stable temperature and humidity were observed until the last 12 h on 29 January when the temperature rose by 10 °C. Temperature increases of this type are common in Calgary due to Chinook winds (the equivalent of Föhn in the Alps), which occur when humid winds blow from the Pacific Ocean east over the Rocky Mountains as described by [14]. The rapid increase in elevation forces cloud buildup on the windward side, combined with rapid temperature decrease, resulting in precipitation over the mountains. The condensation of clouds into precipitation releases latent heat into the air, resulting in a warm wind on the leeward side of the mountains into Calgary and the surrounding area. During this 12 h period, the relative humidity dropped as the saturation point increased with temperature, despite the simultaneous increase in absolute humidity (water vapour pressure). Pressure observations changed of up to 7 hPa during the multi-day test period.

**Figure 8:**
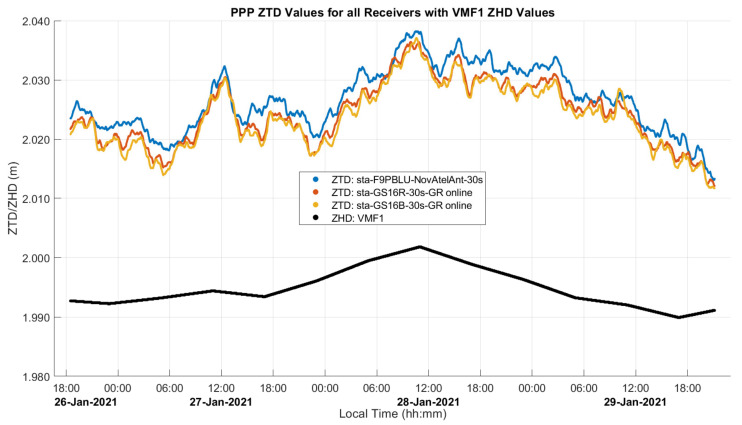
Static dataset A ZTD profiles produced by the three receivers used and VMF1 as initial condition in the software. The three profiles agree within a few millimetres. The ZHD estimated using only VMF1 with the PPP-derived coordinates is also shown; the 6 h update rate of VMF1 is evident. The difference between ZHD and ZTD is ZWD as shown in Figure 9. Local time is UTC−7:00.

**Figure 9:**
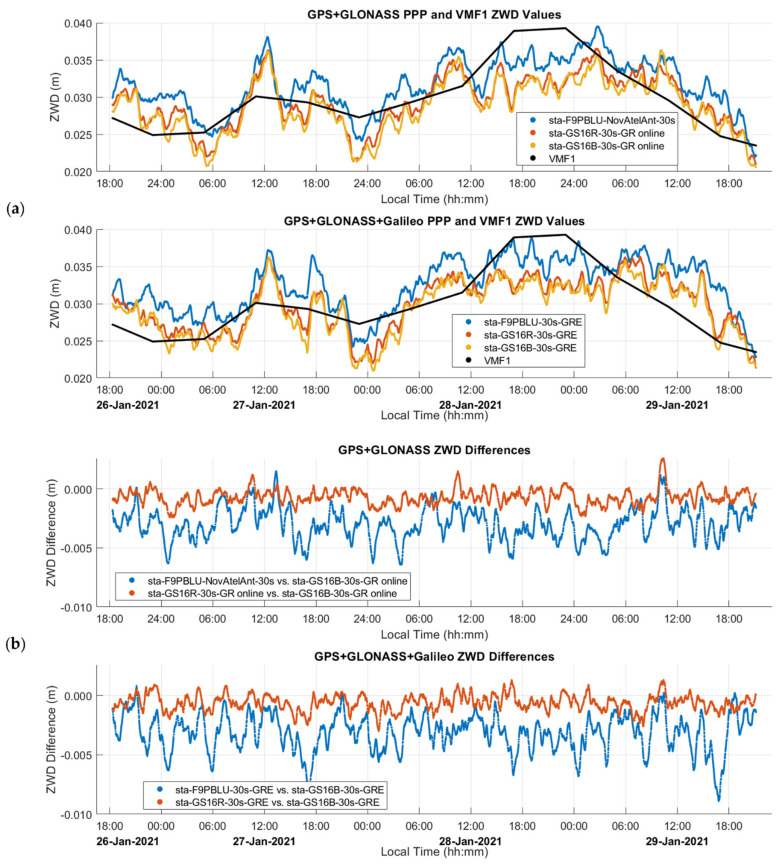
(**a**) Static dataset A ZWD profiles obtained by the three receivers and superimposed by the VMF1 profile for GR and GRE constellations; (**b**) The corresponding ZWD differences between receivers and solutions. Local time is UTC−7:00.

The estimated ZTD and ZWD profiles of this static test are shown in Figure 8 and Figure 9, and comparison statistics are summarized in Table 1 and Table 2. The 2σ uncertainties of the derived profiles are those provided by the PPP software. The GPS and Galileo ambiguity resolution success rate is above 94% in all cases. The three-day continuous measurements were processed as one solution for each receiver. Figure 8 shows the three estimated GR ZTD profiles and the modelled ZHD profile used in the solution as obtained by VMF1 through PPP as discussed previously for Figure 3. The ZTD profiles range from 2.012 to 2.038 m.

Figure 9 shows the ZWD profiles estimated by PPP and VMF1 for both GR and GRE solutions, with ZWD ranging from 20 mm to 40 mm. These values are expected for such a cold winter day at an elevation of over 1000 m. Predictably, the use of three constellations versus two under such open sky conditions does not change the results significantly. The mean difference between GR and GRE solutions is 0.5 mm, with a standard deviation of the difference of 1.7 mm as shown in Table 1. The trend of the PPP-derived profiles is in close agreement with the VMF1 one, the mean difference between the GS16B and VMF1 being less than 1.5 mm and its standard deviation of 3.45 mm as shown in Table 1. The main contribution of GNSS measurements processed in PPP mode in this case is therefore the higher frequency variations which depart from VMF1 by up to about 10 mm.

The agreement between the two GS16 units is at the sub-mm level, both for the mean differences and associated standard deviations, and for both GR and GRE solutions. The absence of antenna calibration for Galileo and different ephemeris products as mentioned earlier did not impact the corresponding solutions in this case. The mean differences between GR and GRE solutions and associated standard deviations shown in the tables remain at the same level as between receivers GR solutions. The number of Galileo satellites available at any time during the solutions varied between 4 and 10.

The quasi-random differences between the two ZWD GS16 profiles are within a few mm and are caused by independent receiver measurement noise and multipath. The same occurs for the GS16B-F9PBLU difference although the noise is slightly higher due presumably to slightly higher F9P carrier phase noise. The F9PBLU final height difference of 17 mm with the GS16B is higher than that for the GS16R as shown in Table 2, and it also exceeds the combined 2σ uncertainties of both height estimates. The ZTD profile of the F9PBLU is offset from the two others by about 3 mm, still within the 2σ values estimated by the software. The difference in height causes the relationship between ZTD and height differences to reach a ratio of 1:7, larger than the ratio of 1:2 suggested by [23]. Two additional static tests also conducted on the CCIT building using the same equipment configuration and similar test durations were analyzed. One test was performed three weeks before test A, while the other was performed one week after; these tests resulted in final height differences of 11.2 mm and 11.8 mm between the F9PBLU and GS16B, with mean ZTD differences of −1.5 mm and −1.8 mm, respectively. The ratio between each of these sets of ZTD and height differences is also 1:7, as above. As the relationship between ZWD and height is consistent between static tests, there is possibly an additional estimated parameter which is highly correlated with height and ZTD, which is contributing to the height difference. The most likely parameter is the receiver clock offset, as it is highly correlated with both ZTD and height [23].

**Figure 10:**
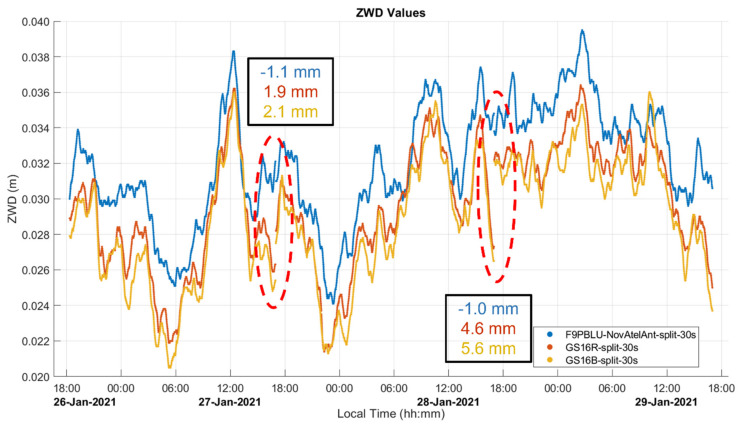
Static Dataset A ZWD profiles split into 24 h segments and differences at the two junction points. Local time is UTC−7:00.

The three-day measurements were also split into three segments of 24 h to determine if agreement at the junction points is similar to that obtained with the IGS PRDS results shown in Figure 4. The results are shown in Figure 10. The differences for the three ZWD profiles range from −1.0 mm to 5.6 mm and are within overall expected ZWD profile accuracy as seen in the 2σ values of Table 1.

A question that arises is the impact of GPS integer versus float ambiguity resolution on ZWD accuracy. The answer is important as integer ambiguity resolution is achieved only part of the time in kinematic mode; even when achieved, it is of short duration due to frequent losses of phase lock on lower elevation satellites caused by obstructions along the road. The question can be partly answered using static data. The GR solutions analysed above already contain GLONASS float ambiguities. GRE solutions processed with the developmental CSRS-PPP were produced for all receivers of test A, using both integer and float ambiguity resolution for GPS and Galileo, and always float ambiguities for GLONASS. RMS differences between ZWD values for integer and float ambiguity solutions reached a maximum of 1.1 mm, with negligible mean differences. GPS only solutions in integer ambiguity mode were also derived. The results were negligibly different from the GR solution, which is not surprising as the addition of GLONASS observations to GR solutions typically results in improved convergence times and robustness, with little effect on solution accuracy, as was discussed in [2]. Hence, it appears that integer ambiguity resolution does not significantly impact ZWD estimation in such long and clean static datasets. Previous studies have shown that for short static datasets of 1 h at globally distributed IGS stations, integer ambiguity resolution improved RMS differences between PPP-estimated ZTD values and reference values provided by the IGS by 33.3%, from 45.7 mm to 30.5 mm [24]. Therefore, for shorter kinematic datasets where integer ambiguity resolution is possible, estimated ZWD profiles may be significantly improved over float ambiguity solutions. However, as mentioned above, integer ambiguity resolution is challenging due to frequent losses of phase lock which are common in typical kinematic trajectories.

**Figure 11:**
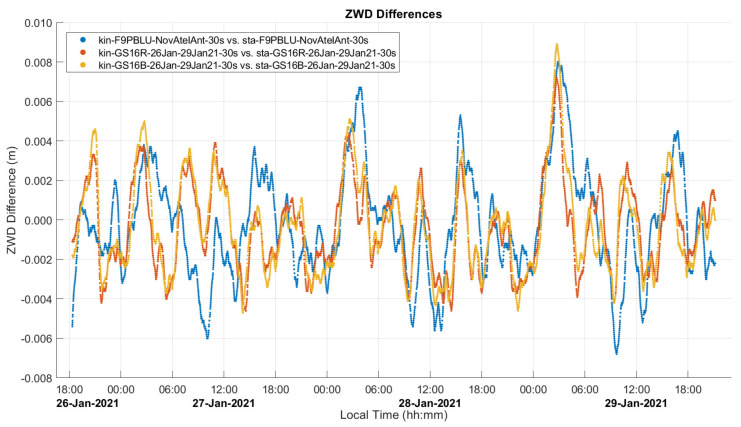
Static dataset A ZWD differences between static and kinematic solutions. Local time is UTC−7:00.

A comparison between static and kinematic solutions of the static data to determine the level of agreement was also conducted and the results are shown in Figure 11. A notable difference between static and kinematic solutions is that in the former, the height is constrained to the final estimated value, while in the latter, the height varies throughout the solution. The range in height variations of the kinematic solutions was on the order of 5 cm for the GS16 units and 10 cm for the F9P. The 2σ uncertainties of the estimated ZWD values are in the range of 3 to 4 mm versus 6 to 8 mm for the static processing case. It is not possible to directly compare 2σ uncertainties of ZWD values between static and kinematic mode because in static mode, there is an additional scaling factor applied to the ZWD uncertainty by the CSRS-PPP software which is not applied in kinematic mode. The 2σ uncertainties of the heights are in the range of 47 to 51 mm versus 3 to 5 mm for the static processing case since in this case, only one height value is estimated from the entire long data sequence. Consequently, in static mode, there are higher frequency variations in ZWD values than in kinematic mode, which exhibits smoother ZWD behaviour as some noise is absorbed by noisier height estimates, as mentioned above, due to the difficulty of separating the two parameters. The differences are at the 2 to 3 mm level and below the noise level of receiver measurement noise.

### 3.3. Kinematic Dataset “B” Analysis

**Figure 12:**
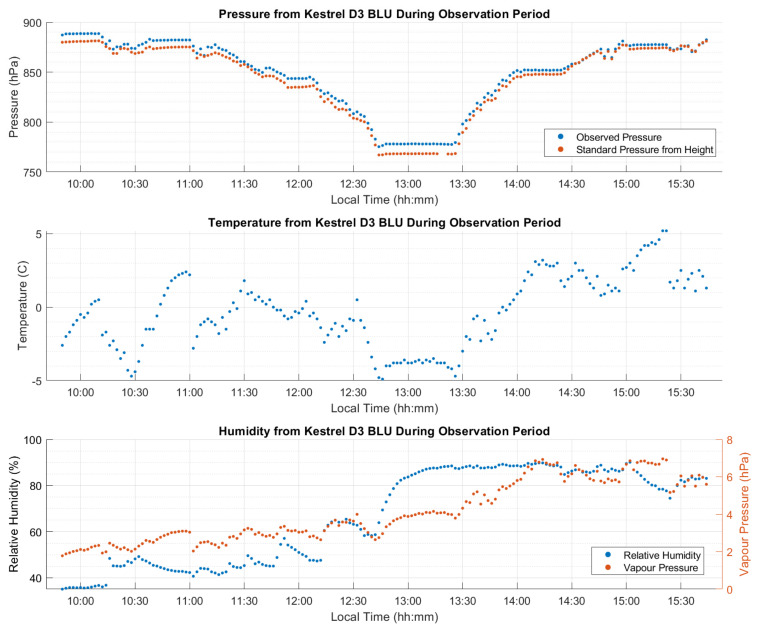
Kinematic dataset B atmospheric measurements made on the vehicle during the test. Measured atmospheric pressure was 5 to 10 hPa higher than standard pressure. Water vapour increased by some 4 hPa during descent due to changing weather conditions. Local time is UTC−6:00.

**Figure 13:**
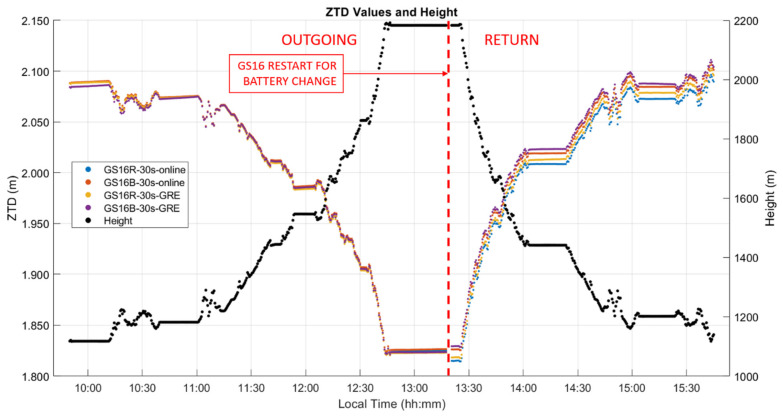
Kinematic dataset B PPP-derived ZTD and height profiles of outgoing and return trajectories. Local time is UTC−6:00.

This test of 290 km was conducted using two GS16 receivers in late October 2020. The atmospheric conditions measured at the vehicle using a Kestrel unit during the six-hour out and back trajectory, which included some stops, the longest one of 40 min at the 2200 m Highwood Pass itself, are shown in Figure 12 and the height and ZTD profiles in Figure 13. The height ascent from the Prairies to the pass was 1100 m. The measured atmospheric pressure was 3 to 10 hPa higher than the normal pressure for the vehicle height throughout the test. A few temperature discontinuities occurred during stops when the Kestrel unit was exposed to insolation without air circulation. The pressure of 768 hPa and temperature of −4 °C while at the pass were relatively stable as conditions became cloudy during this period. Relative humidity however increased rapidly from 60% to 90% while at the pass and remained at that level nearly until the end of the test. These conditions resulted in an increase of water vapour pressure from 3 hPa at the pass to 6 hPa by the end of the test due to the above changing weather conditions. Upon arriving at the pass, measurements continued to be recorded in static mode for 30 min. The two receivers were then turned off to change the internal batteries and restarted. Another five minutes of measurements were recorded in static mode prior to return. The difference between the ZHD profile calculated with Saastamoinen’s model using the above measurements and VMF1 was nearly constant with a −2.9 mm mean difference and associated standard deviation of 4.6 mm; hence, there is no need to show this in a separate figure. Maximum differences between Saastamoinen’s and VMF1 ZHD models reach a magnitude of 15 mm, approximately twice that of the static test A shown above. This increase highlights the challenges of ZHD modelling in mountainous regions with sparsely distributed permanent weather observing stations and the effect of topography on air circulation. As mentioned above, ZHD modelling errors are compensated for by ZWD estimation, thus reducing the effect on position estimates, but still affecting ZWD accuracy for other uses such as in NWP models.

**Figure 14:**
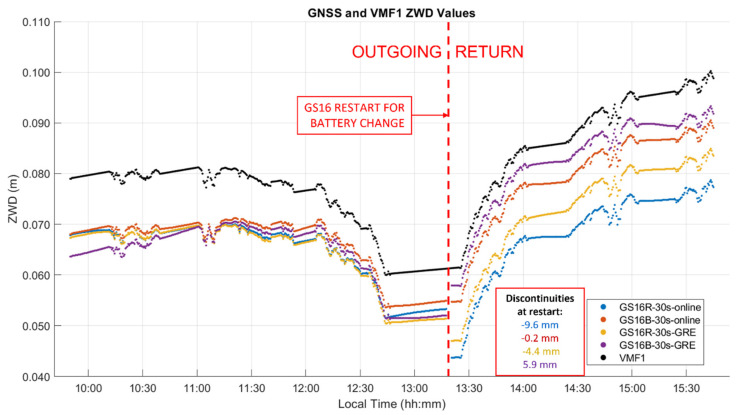
Kinematic dataset B ZWD profiles of outgoing and return trajectories. Local time is UTC−6:00.

**Figure 15:**
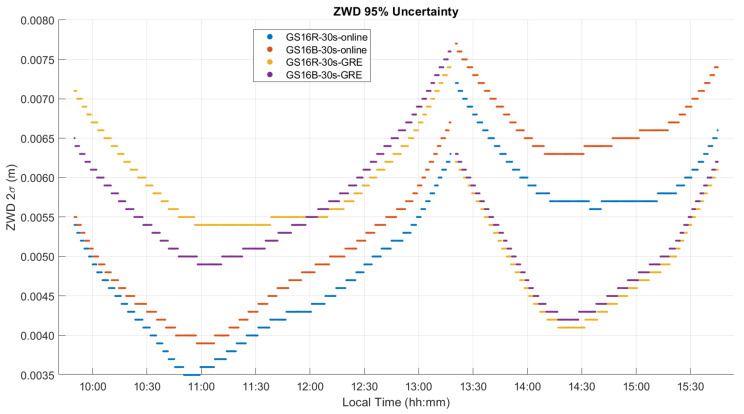
Kinematic dataset B ZWD profile 2σ uncertainties.

The ZTD differences between receivers are the same as those for the corresponding differences between ZWD values shown in Figure 14. The corresponding statistics for ZWD and height differences are given in Table 3 and Table 4. The GPS integer ambiguity resolution success rate ranges from 72% to 93%, a significant decrease as compared to the static case. Integer ambiguity resolution success rates for GRE solutions on the outgoing trajectory are shown as 0.00% in Table 3; this is likely due to the precise products used in GRE processing (GFZ orbit and clock products/CNES observable specific biases), not the addition of Galileo. This effect was investigated by processing GR solutions for the outgoing trajectory using GFZ ephemeris, which also resulted in zero integer ambiguity resolution success. The mean 2σ values are the averaged 2σ values output at each epoch by the software for the entire test. These values are largely determined by the process noise in the PPP filter. However, they are also affected significantly by the short duration of each data sequence. This is shown in Figure 15, where, predictably, the lowest uncertainties of 3 to 4.5 mm are in the middle of each sequence where the smoothing has maximum effect. The uncertainties at the extremities reach 7.7 mm. Nevertheless, this stability also shows that sufficient measurements with appropriate satellite geometry were available during each segment. The discontinuities of the ZWD profiles after the receivers are restarted at the pass range from −0.2 mm for the GS16B to 9.6 mm for the GS16R. The discontinuities for each solution are of different magnitudes and in different directions, eliminating the possibility that there is a consistent systematic effect on all solutions. These initial discontinuities remain nearly constant during the return trajectory, resulting in a mean difference of 11.2 mm between the GS16R and GS16B receivers on the return trajectory, as given in Table 3, versus the mean difference of 1.5 mm on the outgoing trajectory. If one considers that the 2σ estimated ZWD accuracy of one profile is 6 to 7 mm prior and after the data interruption, the maximum difference of −9.6 mm shown in Figure 14 is within the limit of the 2σ values of the difference between the two profiles. This shows the accuracy limit of PPP-derived ZWD kinematic profiles under the type of conditions encountered during the test. Dataset C will confirm the same level of performance.

#### 3.3.1. Kinematic ZWD A-Priori Modelling and Process Noise Analysis

**Figure 16:**
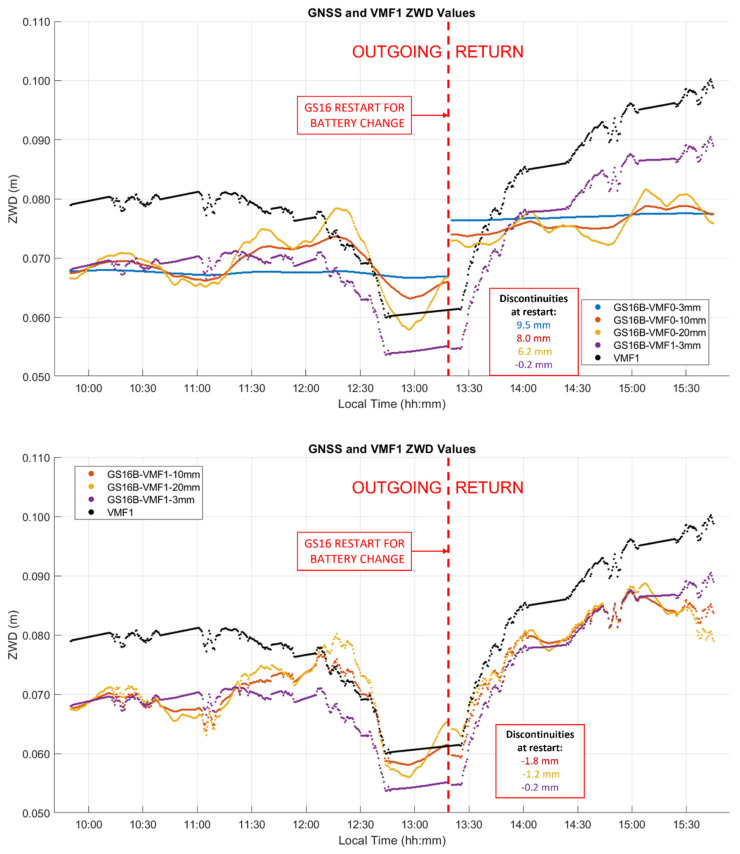
Kinematic dataset B GS16B receiver comparison of PPP ZWD solutions using a constant value of zero as the ZWD a priori input (upper plot, denoted as VMF0) and the VMF1 model as the ZWD a priori input (lower plot), with different levels of process noise. VMF1 model values and PPP solution using VMF1 model as a priori with 3 mm/√hr of process noise are included in both plots. Local time is UTC−6:00.

**Figure 17:**
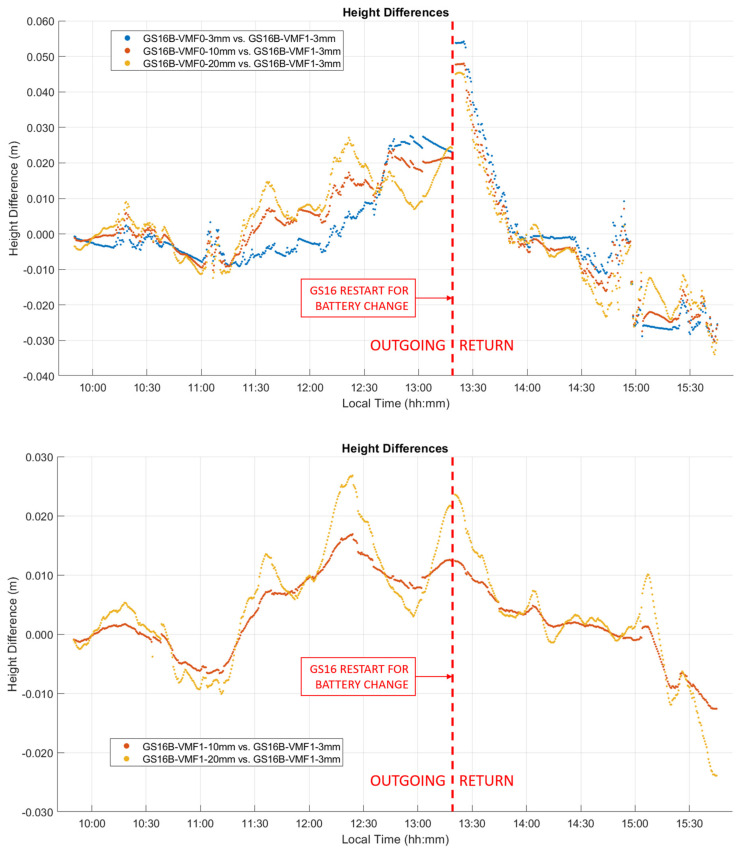
Kinematic dataset B GS16B receiver comparison of PPP height solutions using a constant value of zero as the ZWD a priori input (upper plot) and the VMF1 model as the ZWD a priori input (lower plot), with different levels of process noise. VMF1 model values and PPP solution using VMF1 model as a priori with 3 mm/√hr of process noise are included in both plots. Local time is UTC−6:00.

As seen in Figure 14, the ZWD profiles estimated by PPP closely follow the VMF1 profile, resulting in a standard deviation of 1–2 mm in the difference between the GS16B and VMF1 profiles. To ascertain that VMF1 used initially as input to CSRS-PPP does not overconstrain the output of the latter, the GS16B measurements were re-processed with different ZWD models. Using the developmental PPP software with the same GR constellations and precise products as the online version, solutions were computed with a priori ZWD values of zero (denoted as VMF0) and process noise of 3 mm/√hr (identical to the online software), 10 mm/√hr, and 20 mm/√hr. The developmental software was also used to obtain solutions with the VMF1 a priori ZWD model as carried out in the online version (denoted as VMF1) but with the above 3 mm/√hr, 10 mm/√hr, and 20 mm/√hr process noise. ZWD profiles for all of the above solutions are shown in Figure 16, with statistics of differences with the online solution given in Table 5. The baseline results when using the VMF1 a priori model and 3 mm/√hr process noise are identical to the GS16B online solution shown in Figure 14. For solutions not using the VMF1 a priori ZWD values, it is evident that with 3 mm/√hr process noise, there is no significant correlation in ZWD with height as is seen in the VMF1 model and solutions computed using the VMF1 a priori values. However, when the process noise is increased to 10 mm/√hr, the ZWD profile reacts to changes in height somewhat similarly to the VMF1 model. The changes are much smoother than solutions which use the VMF1 a priori model, as the sharp variations in these solutions are produced by the height corrections for VMF1 grid values to the receiver height derived by [18], as mentioned earlier. When the process noise is increased to 20 mm/√hr, the variation increases further compared to the solution using the VMF1 a priori model. At several points, the 20 mm/√hr solution changed in the opposite direction as was expected for the given height change, e.g., at the periods beginning at 11:15, 12:00 and 14:30 local time. In the first two cases, the receiver height increases, causing the VMF1 model to decrease; however, the estimated ZWD increases. In the third case, at 14:30, the same effect is seen, with the height change occurring in the opposite direction. To determine if this variation reflects actual ZWD variation or if it is simply random instability, the same analysis was performed on the GS16R receiver. The deviations from the VMF1 height corrections of the GS16R results occurred at different times, in different directions and with lower magnitudes. Additionally, the GS16R profiles showed stronger correlation with the height correction variations. As the variations between the two receivers were not similar, it appears that the deviations from the height corrections used in the online solution are more likely to be random instability than true ZWD variations. Therefore, while the ZWD solution variations are strongly constrained to the height corrections applied to the VMF1 model in kinematic mode, it does appear that the height correction method is appropriate and maintains stability in the ZWD solution. The height differences between solutions without an a priori ZWD model and the online solution shown in Figure 17 reach over 50 mm, which is similar to the level of height 2σ uncertainties shown in Table 4. Even when the VMF1 a priori is used in all solutions, the differences with the online solution reach over 25 mm with different process noise; therefore, the impact is significant on PPP kinematic positioning capability at that level of accuracy.

**Figure 18:**
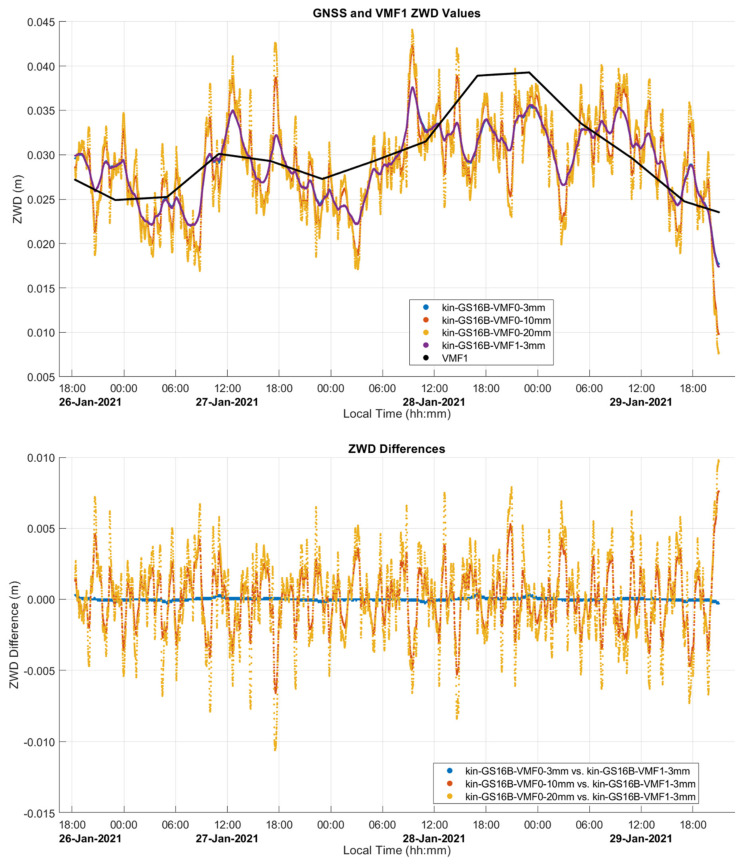
Static dataset A GS16B receiver comparison of PPP ZWD profiles using the VMF1 model in the online software versus a value of 0 with different process noise (upper plot) and differences with respect to online solution (lower plot).

As a comparison, the same analysis was performed with static dataset A processed in kinematic mode and shown in Figure 18. These results show that the VMF1 a priori model does not over constrain ZWD values in static mode, as the solutions with 3 mm/√hr of process noise are nearly identical, except for sub-mm deviations at the 6 h intervals of the VMF1 model. Solutions processed with increased process noise simply increase the noise of the ZWD parameter and again introduce additional noise into height estimates due to the correlation of the two parameters. Hence, 3 mm/√hr is an appropriate ZWD process noise setting in static mode.

### 3.4. Kinematic Dataset “C” Analysis

**Figure 19:**
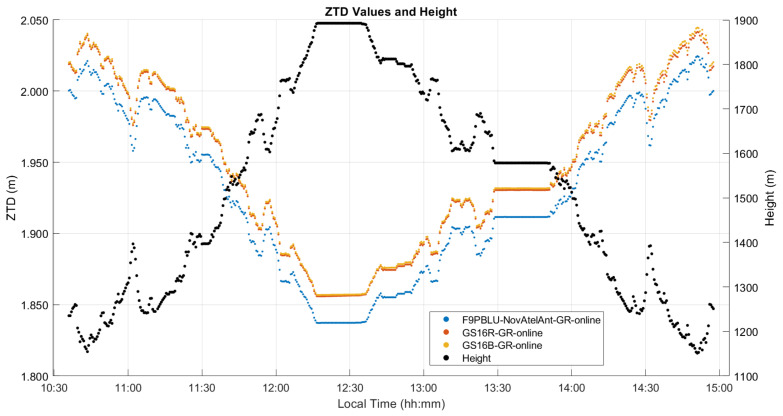
Kinematic dataset C PPP-derived ZTD and height profiles of full trajectory. Local time is UTC−6:00.

**Figure 20:**
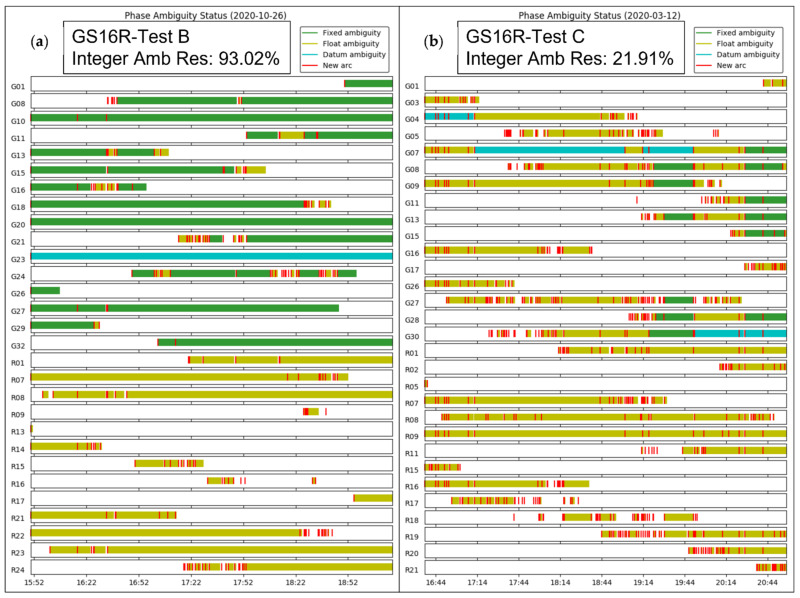
Ambiguity status plot from CSRS-PPP solution report from GS16R solutions for kinematic tests B outgoing only-(**a**) and C (**b**). Time shown is UTC.

**Figure 21:**
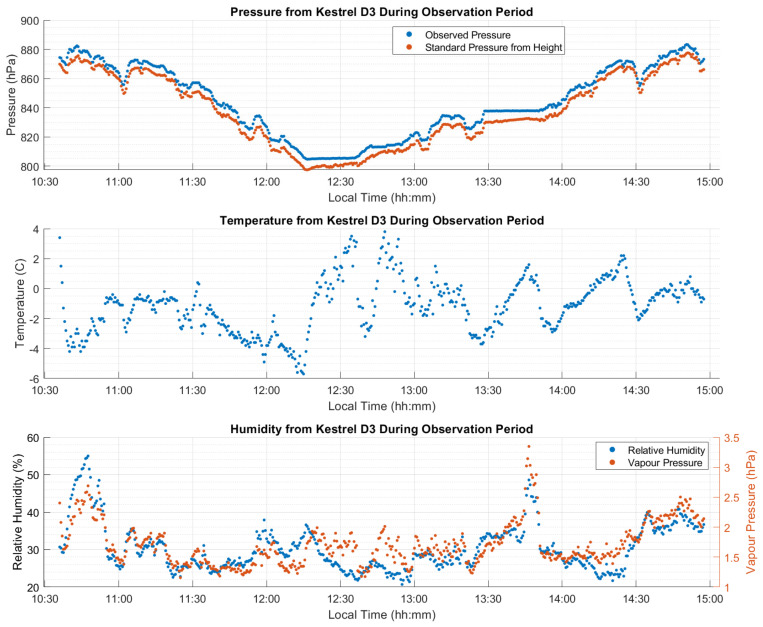
Kinematic dataset C atmospheric measurements made on the vehicle during the test. Measured atmospheric pressure was 3 to 9 hPa higher than standard values. Water vapour spiked at 13:30 local time due to a brief snowfall. Local time is UTC−6:00.

The trajectory for this test is similar in both length and elevation gain to the trajectory of test B, with 280 km travelled beginning in Calgary at 1200 m and going out and back to Chester Lake to a maximum elevation of 1900 m, and with several static stops along the trajectory. The height profile, along with the PPP-estimated ZTD values for each receiver are given in Figure 19 showing a total vertical gain of 750 m. The major difference in this test is that the signal obstructions are significantly higher than those of test B, including several overpasses along the highway and steeper mountains combined with 20 to 30 m conifer trees along the road. The effect of these obstructions can be seen in the ambiguity resolution status plot produced by the CSRS-PPP service shown in Figure 20 for both kinematic tests B and C for the GS16R receiver. There are clearly many more obstructions in test C resulting in breaks in carrier phase tracking and preventing integer ambiguity resolution. No GS16 battery changed occurred at the return point. As with test B, the atmospheric conditions along the road test are shown in Figure 21, consisting of pressure, temperature, and relative humidity measurements. Differences in observed pressure and standard pressure from height range from 3 hPa to 9 hPa, similar to test B. Differences between the ZHD Saastamoinen’s model using these pressure observations and the VMF1 ZHD model resulted in a larger mean difference than in test B at 4.3 mm (versus −2.9 mm in test B), but with a lower standard deviation at 2.4 mm (4.6 mm in test B). The maximum difference was also lower at 10 mm (−15 mm in test B). Temperature variations typically coincided with elevation changes, again with the exception of increases during static stops when the Kestrel unit was exposed to insolation without airflow. A spike in relative humidity and water vapour pressure is seen at 13:50 local time, coinciding with a local snowfall during a stop. Relative humidity then decreased as the vehicle left the area.

**Figure 22:**
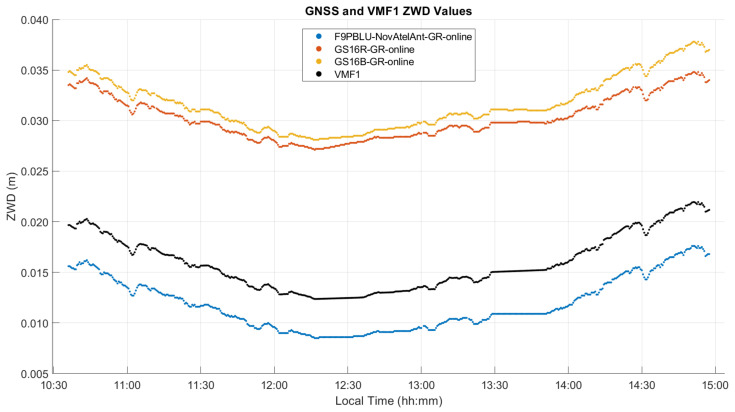
Dataset C ZWD profiles for full trajectory. Local time is UTC−6:00.

The ZWD profiles estimated during the test with the online software are shown in Figure 22 for all receivers and for the VMF1 model. Corresponding statistics for ZWD and height differences are given in Table 6. Increased signal obstructions during the test compared to B resulted in very low integer ambiguity resolution success rates, with no ambiguities resolved except for 22% in the case of the GS16R. Mean 2σ values increased from test B by a factor of 2 to 10 mm for the GS16R and 14 mm for the GS16B, and by a factor of 4 to 19 mm for the F9PBLU. While the mean difference between GS16 receivers does not reflect this increase in uncertainty at 1.4 mm, the mean difference between the F9PBLU and GS16B did increase to 19.8 mm. Standard deviations of the differences between the receivers were at the sub-mm level. Height estimates from PPP were degraded significantly in this test compared to test B, with mean differences, standard deviations of differences, and mean 2σ values all increasing from several centimetres in test B to several decimetres. The cause for the higher degradation of height estimates than ZWD estimates is due to the much smoother constrained process modelling for ZWD, which allows for smoother ZWD estimation despite the much higher noise in height estimates. 

**Figure 23:**
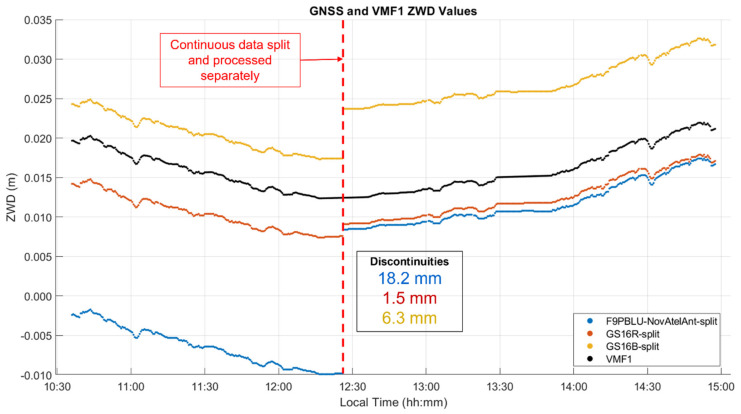
Dataset C ZWD profile with data split in the middle. Local time is UTC−6:00.

To further analyze the behaviour of ZWD profiles between separate segments, an analysis similar to that of test B was performed. Halfway through the static stop at the Chester Lake turnaround point, the measurement sequence was split into two segments before separate PPP processing. This allowed for the discontinuities of the PPP estimation process to be examined. There was an overlap of one epoch between segments to eliminate the 30 s gap in epochs. The ZWD profiles estimated for both segments are shown in Figure 23, with corresponding ZWD difference statistics between receivers in Table 7. The discontinuities at the split, given in the figure, range from 1.5 mm for the GS16R to 18.2 mm for the F9PBLU. These discontinuities are highly dependent on the epoch at which the data is split, however. When the same procedure is performed with the data split at the beginning of the turnaround static stop rather than the middle, the discontinuity for the GS16R is 17.6 mm, while the F9PBLU is 1.6 mm, even lower than the GS16B at 4.6 mm. Interestingly, the integer ambiguity resolution for the GS16B was 17% for the return segment, despite being 0% for both the outgoing segment and the full trajectory, while the success rate was 19% for the GS16R on the return, and also 0% on the outgoing segment. Each of the GS16 return segments also had 2σ values approximately 0.5 times the magnitude of their outgoing segments, reducing from 31 mm on the outgoing for both to 15 mm and 13 mm on the return for the GS16R and GS16B, respectively. These results show the instability of the PPP zenith delay estimation process under such harsh signal availability conditions and therefore the limitation of kinematic profiling.

In each of the cases described above for test C, both the differences in estimated ZWD values for different receivers and the discontinuities introduced by data segment splitting fall within the overall uncertainty of ZWD estimates given by the 2σ values output by PPP. Therefore, these 2σ values provide a realistic evaluation of the accuracy that can be expected from estimated ZWD values in PPP. The results of this test show that higher levels of GNSS signal obstruction do increase the uncertainty of ZWD estimates significantly, in this case by a factor of two to four depending on the receiver. Uncertainty estimates are also affected by the length of the trajectory and can be affected by the inclusion or exclusion of specific segments of data, as is shown by the difference in GS16B and GS16R uncertainty estimates and integer ambiguity resolution success between the outgoing and return segments of the test.

## 4. Conclusions

The concept of using GNSS-PPP to estimate ZTD profiles and components in kinematic mode in sub-optimal signal line-of-sight conditions encountered on mountain roads has been demonstrated, although with slightly lower performance than that of the static case. The approach, however, becomes ineffective when signal obstructions due to a combination of steep slopes and forests along roads degrade observability. In both cases, the absence of long segments of continuous carrier phase measurements caused by signal obstructions affects performance. Estimated discontinuities caused by the latter reach 10 to 20 mm when using the high-grade Leica GS16 receivers. Performance of the u-blox ZED-F9P receiver with a geodetic antenna was found to be comparable to that of the high-grade GS16, but with a larger disagreement in ZWD values (3 mm in the ideal static case). The CSRS-PPP software used to obtain profiles was first tested using IGS station 24 h static data sets as a reference. This produced ZTD values in agreement at the level of 5 mm in RMS difference with the IGS derived values, demonstrating its capability. The estimated two-sigma values of the profiles estimated by the software were found to be realistic and discrepancies between receivers used during the tests were within these error bounds. The addition of Galileo did not improve performance significantly in the cases tested, signal geometry and continuous carrier phase measurement limitations being the overwhelming factors. The VMF1 performance to model the variable ZTD profiles was found to be realistic, especially in its ability to account for large height variations during kinematic testing. The main contributions of GNSS PPP are then to provide three-dimensional coordinates to the model, estimate a near constant bias of up to a few cm and produce a more detailed profile resolution between the six-hour updates of the model. The tests were conducted at elevations of between 1000 to 2200 m, at temperatures ranging from −15 to 5 ˚C and relative humidity of 40 to 90%. ZTD values ranged from 1.8 to 2.2 m depending largely on elevation. The ZWD parts ranged from a few to 10 cm during the winter tests presented. The RMS differences between the GNSS-PPP-derived ZWD and VMF1 profiles were of the order of 4 mm for the static baseline test case and 9 and 16 mm for the two kinematic profiles analyzed, which is the estimated accuracy of the GNSS-PPP derived profiles. An important limitation in kinematic mode is the higher height variation and errors between points, which results in lower estimated ZWD accuracy due to the high correlation between the two. If accurate ZWD estimation is required for numerical weather modelling purposes, the additional factor of ZHD modelling errors must be considered. The difference between VMF1 and Saastamoinen’s model (using pressure observations at the receiver) reached values of 5 to 15 mm, which almost directly translates to additional ZWD error. Hence, the use of atmospheric pressure observations at the receiver is highly beneficial for this purpose. An interesting final observation that applies to the kinematic profiles described in the paper is that, if one were interested only in the VMF1 model to obtain a good approximation of the ZWD profile, the coordinates needed to generate this profile could be provided by Android phones capable of collecting single-frequency raw GNSS measurements. The accuracy of such coordinates varies between sub-metre in open roads to a few metres in harsh signal reception [25], which is sufficient to derive a VMF1 profile.

Finally, a method to further study the troposphere along mountainous profiles and supplement vehicular measurements would be the use of drones equipped with precise GNSS equipment and meteorological instruments. A drone flying over the vehicle 30 to 50 m would deal with the signal obstruction limitations encountered in the current study. The height difference would be accurately known and its impact on ZTD difference easily accounted for. Alternatively, the drone could completely replace the vehicle. The use of more than one drone flying the same trajectory at different elevations above the ground would allow one to study vertical ZTD profiles with a high level of resolution.

## Figures and Tables

**Table 1:** Static Dataset A ZWD profile mean differences, standard deviations and average 2σ values for GR, GRE, and VMF1, and integer ambiguity resolution success rates.

Processing Mode	Receiver (Differenced vs. GS16B)	Mean Difference (mm)	STD DEV Difference (mm)	Mean 2σ Uncertainty (mm)	Integer Ambiguity Resolution (%)
GR (online)	F9PBLU	−2.95	1.35	8.22	94.14
	GS16R	−0.69	0.67	7.68	99.87
	GS16B	-	-	7.68	99.70
	VMF1 ZWD	−1.40	3.45	-	-
GRE (developmental)	F9PBLU	−3.13	1.56	7.03	95.55
	GS16R	−0.62	0.67	6.93	95.25
	GS16B	-	-	6.94	94.11
	VMF1 ZWD	−0.81	3.46	-	-
GRE vs. GR	F9PBLU	−0.89	1.95	-	-
	GS16R	−0.52	1.72	-	-
	GS16B	−0.59	1.70	-	-

**Table 2:** Static dataset A height final differences and 2σ uncertainties.

Processing Mode	Receiver (Differenced vs. GS16B)	Final Difference (mm)	Final 2σ Uncertainty (mm)
GR (online)	F9PBLU	17.30	5.20
	GS16R	3.20	4.60
	GS16B	-	4.60
GRE (developmental)	F9PBLU	16.80	3.80
	GS16R	2.60	3.70
	GS16B	-	3.70
GRE vs. GR	F9PBLU	1.90	-
	GS16R	1.80	-
	GS16B	2.40	-

**Table 3:** Kinematic dataset B ZWD profile mean differences, standard deviations, and average 2σ uncertainties for GR, GRE, and VMF1, and integer ambiguity resolution success rates.

Processing Mode	Receiver (Differenced vs. GS16B)	Mean Difference (mm)	STD DEV Difference (mm)	Mean 2σ Uncertainty (mm)	Integer Ambiguity Resolution (%)
Outgoing trajectory					
GR (online)	GS16R	1.54	0.82	4.50	93.02
	GS16B	-	-	4.83	89.11
	VMF1 ZWD	−8.74	1.96	-	-
GRE (developmental)	GS16R	0.02	1.73	5.95	0.00 ^1^
	GS16B	-	-	5.74	0.00 ^1^
	VMF1 ZWD	−10.72	2.49	-	-
GRE vs. GR	GS16R	0.45	0.55	-	-
	GS16B	1.98	1.18	-	-
Return trajectory					
GR (online)	GS16R	11.22	0.52	6.02	78.68
	GS16B	-	-	6.71	72.71
	VMF1 ZWD	−8.03	0.98	-	-
GRE (developmental)	GS16R	9.62	0.81	4.89	74.17
	GS16B	-	-	4.99	74.27
	VMF1 ZWD	−4.69	1.26	-	-
GRE vs. GR	GS16R	−4.95	1.08	-	-
	GS16B	−3.34	0.43	-	-

^1^ GR solutions using GFZ ephemeris products also resulted in 0% integer ambiguity resolution success; hence, there was likely no issue with the addition of Galileo.

**Table 4:** Kinematic dataset B height profile mean differences, standard deviations, and integer ambiguity resolution success rates for GR and GRE.

Processing Mode	Receiver (Differenced vs. GS16B)	Mean Difference (mm)	STD DEV Difference (mm)	Mean 2σ Uncertainty (mm)	Integer Ambiguity Resolution (%)
Outgoing trajectory					
GR (online)	GS16R	−27.49	26.78	47.79	93.02
	GS16B	-	-	53.07	89.11
GRE (developmental)	GS16R	−10.63	32.98	48.52	0.00
	GS16B	-	-	47.84	0.00
GRE vs. GR	GS16R	−7.79	18.79	-	-
	GS16B	−24.65	43.61	-	-
Return trajectory					
GR (online)	GS16R	−37.84	28.89	57.92	78.68
	GS16B	-	-	58.22	72.71
GRE (developmental)	GS16R	−33.26	28.93	41.98	74.17
	GS16B	-	-	41.95	74.27
GRE vs. GR	GS16R	5.12	13.20	-	-
	GS16B	0.54	17.17	-	-

**Table 5:** Kinematic dataset B ZWD profile mean differences, standard deviations, average 2σ uncertainties, and integer ambiguity resolution success rates using different ZWD models.

Segment	ZWD Model Used	Mean Diff vs. VMF1–3 mm (mm)	STD DEV Diff vs. VMF1–3 mm (mm)	Mean 2σ (mm)	Integer Ambiguity Resolution (%)
Outgoing	VMF0–3 mm	−1.37	5.53	4.87	89.91
	VMF0–10 mm	−2.66	4.59	8.74	88.41
	VMF0–20 mm	−2.91	4.61	12.07	88.41
	VMF1–10 mm	−2.23	3.24	8.74	88.41
	VMF1–20 mm	−2.85	4.42	12.07	88.41
	VMF1–3 mm	-	-	4.83	89.10
Return	VMF0–3 mm	2.19	8.68	5.66	77.69
	VMF0–10 mm	3.10	7.77	9.81	74.84
	VMF0–20 mm	3.32	7.44	13.37	74.84
	VMF1–10 mm	−0.33	2.50	10.19	72.71
	VMF1–20 mm	−0.58	3.78	13.81	72.71
	VMF1–3 mm	-	-	6.71	72.71

**Table 6:** Dataset C ZWD and profile mean differences, standard deviations, average 2σ uncertainties, and integer ambiguity resolution success rates for full trajectory.

Parameter	Receiver (Differenced vs. GS16B)	Mean Difference (mm)	STD DEV Difference (mm)	Mean 2σ Uncertainty (mm)	Integer Ambiguity Resolution (%)
ZWD	F9P-BLU	19.81	0.39	19.41	0.00
	GS16R	1.39	0.58	10.12	21.91
	GS16B	-	-	13.77	0.00
	VMF1 ZWD	15.72	0.32	-	-
Height	F9P-BLU	−168.52	766.87	488.73	-
	GS16R	−220.55	664.07	254.76	-
	GS16B	-	-	287.10	-

**Table 7:** Dataset C ZWD and profile mean differences, standard deviations, average 2σ uncertainties and integer ambiguity resolution success rates for split outgoing and return trajectories.

Segment	Receiver (Differenced vs. GS16B)	Mean Difference (mm)	STD DEV Difference (mm)	Mean 2σ Uncertainty (mm)	Integer Ambiguity Resolution (%)
Outgoing	F9P-BLU	26.90	0.21	37.52	0.00
	GS16R	10.08	0.11	30.92	0.00
	GS16B	-	-	31.40	0.00
Return	F9P-BLU	15.21	0.06	31.25	0.00
	GS16R	14.39	0.19	14.70	18.93
	GS16B	-	-	12.68	17.00

## Data Availability

The GNSS measurements collected during the tests presented in the paper can be made by contacting the primary author.
